# STSN-Net: Simultaneous Tooth Segmentation and Numbering Method in Crowded Environments with Deep Learning

**DOI:** 10.3390/diagnostics14050497

**Published:** 2024-02-26

**Authors:** Shaofeng Wang, Shuang Liang, Qiao Chang, Li Zhang, Beiwen Gong, Yuxing Bai, Feifei Zuo, Yajie Wang, Xianju Xie, Yu Gu

**Affiliations:** 1 Department of Orthodontics, Beijing Stomatological Hospital, Capital Medical University, Beijing 100050, China; 2939108747@ccmu.edu.cn (S.W.); changqiao10@sina.com (Q.C.); zhangli_cpums@163.com (L.Z.); gongbeiwen@outlook.com (B.G.); byuxing@ccmu.edu.cn (Y.B.); 2 School of Biomedical Engineering, Capital Medical University, Beijing 100069, China; shliang@ccmu.edu.cn; 3 Laboratory for Clinical Medicine, Capital Medical University, Beijing 100069, China; 4 Beijing Key Laboratory of Fundamicationental Research on Biomechanics in Clinical Application, Capital Medical University, Beijing 100069, China; 5 LargeV Instrument Corp., Ltd., Beijing 100084, China; zuofeifei@largev.com (F.Z.); wangyajie@largev.com (Y.W.)

**Keywords:** tooth segmentation, tooth numbering, instance segmentation, deep learning

## Abstract

Accurate tooth segmentation and numbering are the cornerstones of efficient automatic dental diagnosis and treatment. In this paper, a multitask learning architecture has been proposed for accurate tooth segmentation and numbering in panoramic X-ray images. A graph convolution network was applied for the automatic annotation of the target region, a modified convolutional neural network-based detection subnetwork (DSN) was used for tooth recognition and boundary regression, and an effective region segmentation subnetwork (RSSN) was used for region segmentation. The features extracted using RSSN and DSN were fused to optimize the quality of boundary regression, which provided impressive results for multiple evaluation metrics. Specifically, the proposed framework achieved a top F1 score of 0.9849, a top Dice metric score of 0.9629, and an mAP (IOU = 0.5) score of 0.9810. This framework holds great promise for enhancing the clinical efficiency of dentists in tooth segmentation and numbering tasks.

## 1. Introduction

Tooth segmentation and numbering are of immense significance in dental care, owing to their critical role in assessing oral health and facilitating precise diagnosis [1]. Panoramic radiograph (PR), a two-dimensional (2D) X-ray that captures the entire mouth in a single image, is commonly used for the clinical diagnosis of tooth segmentation and numbering [2]. Cone beam computed tomography (CBCT) is an imaging technology that provides three-dimensional (3D) images of the teeth, soft tissues, nerve pathways, and bone in a single scan [3]. While CBCT offers superior details and a 3D perspective, panoramic radiographs exhibit advantages such as lower radiation exposure and reduced imaging costs [4]. Furthermore, the latter is more commonly used, owing to its simplicity and effectiveness in general dental evaluations, routine screenings, and initial diagnostic tasks [5]. Panoramic radiography is particularly useful in identifying dental caries and periodontal diseases and assessing tooth orientations, making it a versatile tool in a range of common clinical scenarios [6,7]. Obtaining tooth segmentation and numbering is a critical aspect of dental care and is typically performed by dental professionals to ensure accurate assessment and treatment [8]. Normally, the accurate segmentation and numbering of teeth is difficult and labor-intensive. The task becomes even more complicated when using panoramic dental X-ray images as overlapping boundaries between the teeth pose difficulties in the annotation [9]. In addition, this procedure is often performed manually, which can be quite time-consuming. The segmentation and numbering are not objective, and there is no guarantee of the accuracy of the results as it relies heavily on the subjective judgment of the dentist [10].

With the advancements in image processing technology and artificial intelligence, automatic tooth segmentation and numbering have become feasible and have significantly alleviated the burden of annotation for dentists [11]. Nevertheless, panoramic X-ray images of different patients’ teeth exhibit considerable variations, encompassing diverse pathological presentations and treatment indications [12]. In addition, the background of panoramic X-ray images can be quite complex, incorporating redundant background information, which may lead to incorrect segmentation [13]. The accurate segmentation and numbering of teeth in panoramic X-ray images become quite challenging because of these issues, thus impeding the practical utility of automated intelligent segmentation and numbering methods in clinical settings. To overcome these challenges, there is a critical need to develop advanced image processing techniques that can distinguish teeth from their complex backgrounds and accommodate variations in dental conditions, ultimately enhancing the reliability and applicability of automated dental image analysis tools in real-world clinical scenarios.

The accurate segmentation of objects (including tooth segmentation) from images is a key application of computer vision technology [14] and is the cornerstone of quantitative analysis (for example, numbering) that aims to cluster the pixels of a specific category [15]. Since the development of CNN, the use of conventional segmentation methods has declined [16]. Various CNN-based structures have been proposed and applied in semantic segmentation, of which fully convolutional networks (FCNs) that have an encoder–decoder structure are a milestone [17]. Since then, several types of CNN architectures, such as ResNet [18], ResNext [19], VGG [20], and GoogleNet [21], have been used as encoders and decoders. U-net [22] is based on FCN [23] and exhibits the advantages of good performance, low data requirement, and high speed. Various segmentation methods based on deep convolutional neural networks (CNNs), including crowd counting [24,25], text recognition [26,27], and medical image analysis [3,28,29,30,31], have been proposed and are widely used to obtain statistics on target objects [32,33,34].

To further enhance the clinical utility of panoramic X-rays, the development of automated tooth segmentation and numbering methods has gained considerable attention. Segmentation of individual teeth and their subsequent numbering can streamline the diagnostic process, allowing dentists to rapidly and accurately assess tooth morphology and spatial relationships. This approach is particularly valuable in identifying tooth loss, a critical concern, especially in the transitional dentition phase, where early intervention can mitigate the severity of permanent tooth alignment issues. Moreover, the integration of automated assistance in dental diagnostics substantially reduces the likelihood of diagnostic errors by oral health practitioners. Such automation aids in the meticulous documentation of patient records, thereby enhancing the overall quality of dental care. Elif Bilgir et al. proposed the Faster R-CNN Inception v2 model to predict the label and bounding box of the 2482 panoramic radiographs, with an average accuracy of 0.9652 [8]. Münevver Coruh Kılıc et al. devised a method using Faster R-CNN Inception v2 (COCO) for the automatic detection and numbering of deciduous teeth on pediatric panoramic radiographs based on 421 panoramic images to improve the accuracy of deciduous tooth position recognition [35]. André Ferreira Leite et al. improved the accuracy of tooth segmentation and recognition in curved panoramic radiographs of permanent dentition based on 153 radiographs using two deep CNNs [11]. Sheng improved the accuracy of the detection and localization of tooth tissue on panoramic radiographs using the PLAGH-BH tooth segmentation [36]. However, the above methods treated each tooth as a uniform class and failed to incorporate information on distinct tooth shapes and positions in the analysis framework. Moreover, the features were extracted from entire panoramic radiographs without any prior constraints; thus, the ratio of false positives may be high.

To overcome these problems, the proposed framework presents a two-stage network architecture (named STSN-Net) to simultaneously segment the teeth and number them, innovatively delineating the processes into bone window segmentation and tooth segmentation with numbering. The proposed approach can considerably improve the accuracy of feature learning and efficiently eliminate redundant data. The initial stage employed a graph convolution neural network (GCN) to obtain the structure information hidden in the PRs for precise bone window segmentation. The second stage incorporated a shared 2D CNN, augmented by a dual-stage detection subnetwork (DSN) and a sophisticated refinement segmentation subnetwork (RSSN), adding robustness to the framework. The following are the key contributions of this work:

1. A GCN for automatic bone window segmentation was proposed to generate masks of regions to train the RSNN. This process leveraged dental segmentation annotations for initial calibration, followed by feature extraction and mask refinement via GCN.
2. A modified DSN was proposed, which made use of a dilated convolution module in certain specific layers to enlarge the receptive field and employed a deformable convolution block to compensate for the diversity in tooth morphology and distribution among individuals.
3. The proposed RSSN aimed to accurately segment the tooth region within the images, simultaneously suppressing the background noise and resulting in refined predictions that substantially reduced analytical errors.
4. The high precision and strong practicability of the proposed STSN-Net were demonstrated. The proposed two-stage structure initially deployed a GCN for bone window extraction, followed by a multinetwork structure that performed object detection and refinement segmentation, respectively, and simultaneously. Furthermore, STSN-Net was adeptly optimized and adapted for real-world clinical applications in embedded devices.

The rest of the paper comprises four parts. The materials and methods are described in Section 2. Section 3 summarizes the experimental results. The discussion is presented in Section 4, and the conclusion is presented in Section 5.

## 2. Materials and Methods

### 2.1. Dataset TSNDS

This paper introduces a new dataset, the tooth segmentation and numbering dataset (TSNDS), which comprises 2116 oral panoramic X-ray images from patients with permanent dentition, encompassing 2D imaging data points of all teeth, the mandible, and parts of the maxilla. The initial 116 images were obtained from the publicly available dataset, and the subsequent 2000 images were retrospectively selected from panoramic radiographs acquired at the Beijing Stomatological Hospital between March 2019 and April 2022. These 2000 PRs were produced using a Sirona digital machine (ORTHOPHOS XG 5 DS Ceph, Bensheim, Germany) with standard parameters, operating with tube voltages of 60–90 kV and tube operating currents of 1–10 mA. A default program of the device with a predetermined magnification of 1.9× and a rotation time of 14.1 s was used for X-ray exposures with a resolution of 2440 × 1280, and the PRs were in portable network graphics (PNG) format. During the data processing phase, the original DICOM files inherently contained sensitive patient information, such as name and age. To address privacy issues, a method was adopted for converting the image data into the PNG file format for storage. The dataset covers a wide range of dental conditions as observed in dental radiographs, which can be broadly categorized into two main types: permanent dentition and primary dentition. Within these categories, various conditions may coexist, including missing teeth, dental crowding, impacted teeth, and teeth with fillings or restorations. Annotation was performed using the online tool “Label Studio”, ensuring a consistent and precise approach to data labeling. Two dental practitioners, each with over 15 years of clinical experience, independently annotated the radiographs. To ensure that the annotations were accurate and reliable, a senior dentist with more than 25 years of clinical experience reviewed and revised the data. The finalized annotated dataset was then used in the study. The annotations adhered to the FDI tooth notation system established by the International Dental Federation in 1970. Each image within the dataset included segmentation annotations for individual teeth and bone windows, along with eight types of positional information on the teeth. The dataset was categorized into three parts: a training part (Train), a validation part (Validation), and a test part (Test) using the train–test–split function (TTSF) of the scikit-learn library. The distribution and details of the dataset split are presented in Table 1 and Table 2.

### 2.2. Data Augmentation

**Figure 1:**
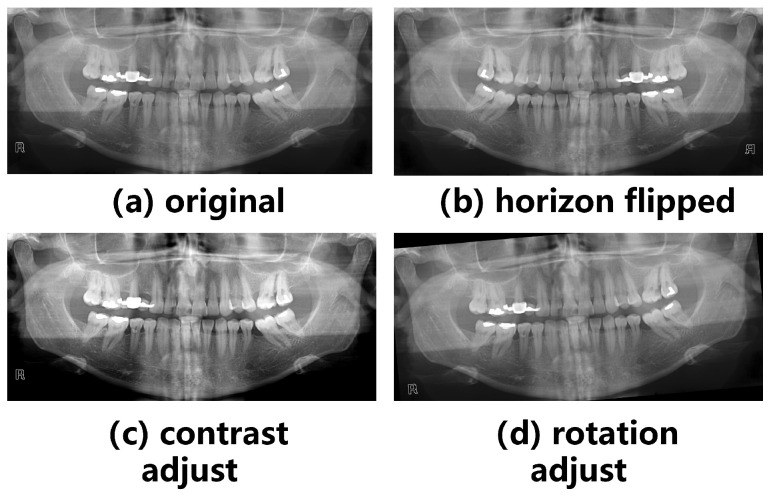
Examples of data augmentation. (**a**) Original image; (**b**) image after horizon flipped augmentation; (**c**) image after contrast adjustment; (**d**) image after rotation adjustment.

The panoramic X-ray images were subjected to a multitude of enhancement processes to augment the dataset while preserving relevant dental features. Via this augmentation pipeline (as shown in Figure 1), diverse transformations such as rotation, scaling, flipping, and contrast adjustments were applied, which facilitated the extraction of robust features and enhanced the ability of the model to generalize. The effectiveness of these augmentation strategies was evaluated and demonstrated via comprehensive experimentation with the section results, showcasing notable improvements in the learning capacity and generalization of the model across diverse dental conditions and variations.

### 2.3. The Pipeline of the Proposed STSN-Net

**Figure 2:**
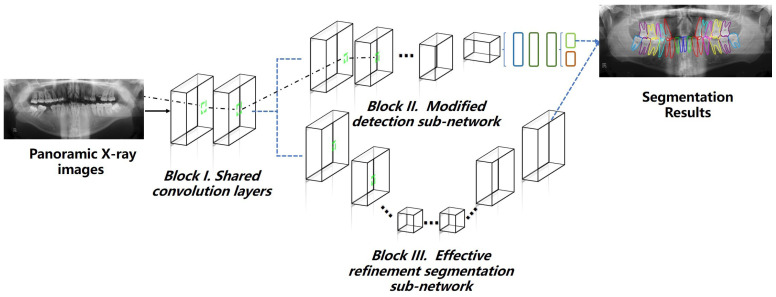
The pipeline of the proposed STSN-Net.

The proposed STSN-Net for simultaneous tooth segmentation and numbering comprised three parts: an automatic annotation method for bone window segmentation, a modified DSN that predicted the bounding boxes and number of teeth, and an RSSN that extracted refinement segmentation information from the regions of interest (as shown in Figure 2). The details of these three parts of the proposed method are described in the following subsections.

### 2.4. Automatic Segmentation of Mandibular Morphology

**Figure 3:**
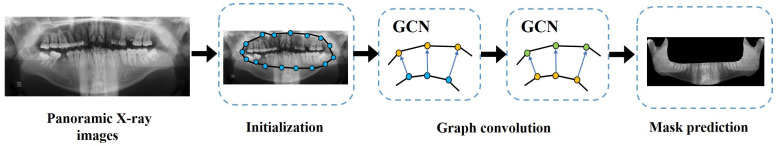
The pipeline of the GCN.

To obtain an accurate boundary annotation of the target area, a novel network architecture was proposed. The annotation of the bounding boxes was used as an initialization. The spatial and gray-level information were subsequently propagated via a multilayer GCN. In the initialization phase, *N* points were selected randomly to form a graph G. The propagation process for a node $g_{i}$ was defined as follows:

(1) $$f_{i}^{\left(n+1\right)}=w_{0}^{n}f_{i}^{n}+\sum_{g_{i}\in N\left(g_{i}\right)}w_{1}^{n}w_{j}^{n}$$

where $w_{0}^{n}$ and $w_{1}^{n}$ represent the weight matrices and $N(g_{i})$ is a set that contains all the nodes connected to node $g_{i}$. The parameters of the pretrained GCN model were fed into the model for rapid convergence. After the initialization, the nodes extracted from the edges of the segmentation annotations were used as target values for mandibular morphology segmentation. The network structure and processing pipeline are illustrated in Figure 3.

### 2.5. STSN-Net Structure

The proposed STSN-Net architecture comprised three blocks (shared convolution layers, a modified DSN, and an RSSN), as shown in Figure 2. Block I represents the two-layer shared convolution layers; 64 convolution filters with a size of 3 × 3 were used in this subnetwork to generate feature maps with 64 channels. The inputs to the shared convolution layers were the PRs, and the output was the feature map named shared features. The extracted shared features, usually identified as shallow characteristics, were concurrently inputted into Block II and III to enhance the efficiency of feature reuse and considerably reduce the overall model complexity and size. Block II was the proposed DSN, which had an improved convolution filter capable of adapting to perspective changes and obtaining a large receptive field. Block III was the proposed RSSN, which distinguished between the foreground and background while generating the instance mask of each tooth from the previous mandibular morphology segmentation generated by the GCN block described in Section 2.4 and shared spatial information on the target region with the DSN via an element-wise sum operation before the RPN in the DSN.

### 2.6. Detection Sub-Network

**Figure 4:**
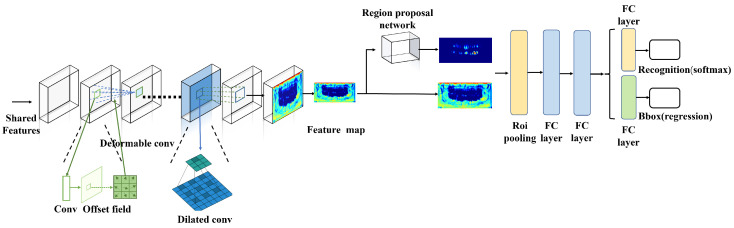
The architecture of the DSN.

The proposed DSN is a modified two-stage detection network. The input of the subnetwork was generated using a two-layer shared convolution block in the STSN-Net structure. The DSN is also a multitask network that has certain shared convolution layers and two parts that perform recognition and bounding box regression. In the shared convolution layers, the normal 3 × 3 convolution filter was replaced with a deformable convolution filter in the second layer to handle the different perspectives. The deformable convolution filter comprised two parts: the convolution and the offset. It is a variant of the normal convolution filter. The formulation of the deformable convolution filter was defined as follows:

(2) $$y\left(p_{0}\right)=\sum_{p_{n}\in R}w\left(p_{n}\right)\cdot x\left(p_{0}+p_{n}+\Delta p_{n}\right)$$

where $p_{n}$ is a location on the feature map y, and $\Delta p_{n}$ is an extra offset added to the input feature map. In this way, the convolution filter extracts features in a deformable manner to handle the changes in the perspective. A dilated convolution filter, which was originally used for semantic segmentation, was employed in the fifth layer to obtain a large receptive field. This filter inserted empty cells into the convolution output. This procedure was defined as follows:

(3) $$f_{n+1}=D\left(f_{n}\right)$$

where $f_{n}$ represents the input feature map of the dilated convolution layer, $f_{n+1}$ represents the output feature map of the dilated convolution layer, and D represents the dilated filter that filters the input feature map by inserting cells with a value of 0 in the convolution output. The filter size was increased from 3 × 3 to 5 × 5 so as to expand the receptive field without adding extra computational burden. The architecture of the proposed DSN is shown in Figure 4.

### 2.7. Refinement Segmentation Sub-Network

**Figure 5:**
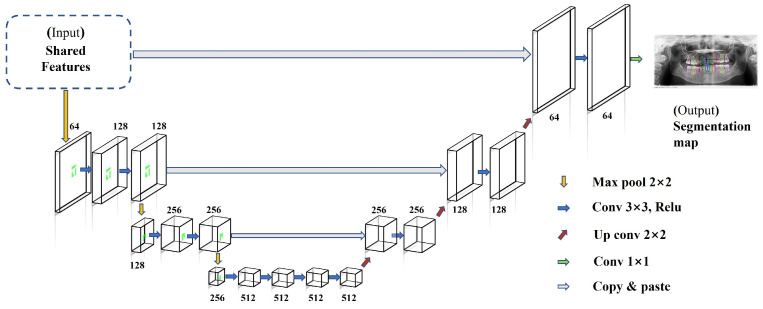
The architecture of the RSSN.

The proposed RSSN is a multiscale CNN-based network that comprised three components: a skip connection component that fused the shallow feature map with the deep feature map, a max pool component that performed downsampling of the high-resolution feature map to obtain global features, and an up-convolution component that restored scale information while generating the segmentation map. As depicted in Figure 5, a max pooling filter with a size of 2 × 2 was adopted for downsampling, a convolution filter with a size of 3 × 3 was employed to extract the features in each subnet, an up-convolution filter was applied to perform upsampling, and the skip connection operation was performed using the copy and paste operation. In the last phase, a 1 × 1 convolution filter was used to reduce the dimension of the feature map and generate the final segmentation map.

### 2.8. Loss Function

The entire framework was trained end-to-end using a batch training method. The loss function of the network consisted of two main parts, defined as follows:

(4) $$L_{\mathrm{all}}=\alpha L_{\det}+\beta L_{\mathrm{seg}}$$

where α and β are the weight parameters of the two tasks. In this study, the values are 0.8 and 0.2, respectively.

The loss of the DSN was defined as follows:

(5) $$L_{\det}=L_{\mathrm{classification}}+L_{\mathrm{reg}}$$

The classification loss of the DSN is a logarithmic loss, which was defined as follows:

(6) $$L_{\mathrm{classification}}=-\log\left(p_{i}\right)$$

where $p_{i}$ represents the probability of the output belonging to class *i*. The regression loss of the DSN was defined by a smooth *L*1 function, as follows:

(7) $$L_{\mathrm{reg}}=\sum_{i\in \{x,y,w,h\}}{\mathrm{smooth}}_{L_{1}}\left(t_{i}^{k}-g_{i}\right)$$

where $t_{i}^{k}$ is a set $x_{i}^{k},y_{i}^{k},w_{i}^{k},h_{i}^{k}$ that forms a bounding box of class *k*, and $g_{i}$ represents the ground truth of the predicted value.

The smooth L1 function was defined as follows:

(8) $${\mathrm{smooth}}_{L_{1}}\left(x\right)=\left\{\begin{array}{cc} 0.5x^{2} & \mathrm{if}\;|x|<1\\ |x|-0.5 & \mathrm{otherwise} \end{array}\right.$$

The segmentation loss is a cross-entropy (CE) loss function, defined as follows:

(9) $$L_{\mathrm{seg}}=\mathrm{CE}\left(m_{p},m_{g}\right)$$

where $m_{p}$ is the predicted segmentation map and $m_{g}$ is the ground truth of the mask map. The CE function for each pixel in the two-class segmentation map was defined as follows:

(10) $$\mathrm{CE}\left(p,\hat{p}\right)=-\left(p\log\left(\hat{p}\right)+\left(1-p\right)\log\left(1-\hat{p}\right)\right)$$

where *p* is the ground truth probability of the target belonging to class 0, and $\hat{p}$ is the prediction probability of the target belonging to class 1. The optimization function was obtained by the stochastic gradient descent (SGD) method.

## 3. Results

### 3.1. Evaluation Metrics

The *F*1 score was used as the evaluation metric because it considers the accuracy and recall of the model and can be regarded as a harmonic average of the model accuracy and recall for tooth numbering. *R* was used to represent recall, and *P* was used to represent accuracy. *R* was defined as follows:

(11) $$R=\frac{TP+TN}{N}$$

*P* is defined as follows:

(12) $$P=\frac{TP+TN}{TP+TN+FP+FN}$$

where *N* represents the number of all targets. *TP* represents the true positive, *TN* represents the true negative, *FP* represents the false positive, and *FN* represents the false negative. True and false were determined by calculating the numbering results and the intersection over union (IOU), which is a metric that compares the similarity and diversity of the detection and segmentation results. The *IOU* was defined as follows:

(13) $$\mathrm{IOU}=\frac{\mathrm{Area}\;\mathrm{of}\;\mathrm{overlap}}{\mathrm{Area}\;\mathrm{of}\;\mathrm{Union}}$$

where the area of overlap is the overlap between the predicted bounding box and the actual bounding box, and the area of union is the union of the predicted bounding box and the ground truth. A threshold parameter was set to determine whether the result was true. If the IOU score was >0.5, it was considered a true detection, otherwise, it was a false detection. The DICE coefficient is a common evaluation metric used in segmentation tasks to measure the accuracy of segmentation results. It quantifies the degree of overlap between the predicted segmentation and the ground truth. The DICE coefficient was defined as follows:

(14) $$DICE=\frac{2\times |A\cap B|}{\left|A|+|B\right|}$$

where *A* is the prediction and *B* is the ground truth. The F1 score was defined as follows:

(15) $$F1-score=\frac{2PR}{P+R}$$

### 3.2. Experiment Results

Multiple experiments were performed to verify the contribution of each part of the proposed method. In our experiments, a two-stage architecture network was adopted, with a backbone network of convolutional neural networks as the baseline. Furthermore, the performance achieved using the multitask learning instance segmentation framework named Mask RCNN [37] and the single-stage detection architecture named YOLOv8 [38] on the dataset was determined for a comprehensive comparison.

Each part of the proposed method was evaluated separately to obtain a better understanding of the proposed method. Common image augmentation operations, including rotation, horizontal flipping, and vertical flipping, were performed to enlarge the training dataset. Network training was performed with two NVIDIA GeForce RTX 4090 GPUs (NVIDIA Corporation: Santa Clara, CA, USA) for 30 epochs. The learning rate was initialized as 0.01, and the factor was decreased by 0.1 after 24 and 29 epochs, respectively. A batch training scheme utilizing the stochastic gradient descent (SGD) optimizer was adopted for training, with a batch size of 16. The base width and height of the images were altered to 1333 and 800, respectively, without changing the aspect ratio. A multiscale training scheme was adopted in the training procedure, with scaling factors of 1.0×, 1.2×, 1.4×, and 1.6×. In the test phase, each scale was evaluated separately, and the same scaling factors were used (Table 3). F1 represents the F1 score as defined in Section 3.1. The following test results were obtained using a single GPU (4090). The experimental results are presented in Table 3.

**Figure 6:**
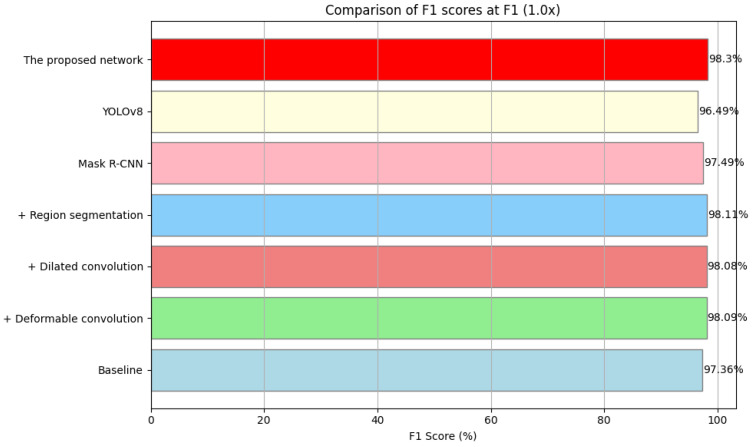
The F1 score comparison.

The *F*1 score measures the harmonic mean of precision and recall, reflecting the accuracy and robustness of the model in detecting instances (as shown in Figure 6). Furthermore, it is imperative to focus on instance segmentation metrics, including mAP (mean average precision) and DICE (Dice similarity coefficient). The mAP metric evaluates the overall detection accuracy across multiple object categories, whereas DICE primarily concerns pixel-level similarity in image segmentation tasks. The experimental results of mAP metrics (IOU = 0.5) and DICE scores are presented in Table 4.

**Figure 7:**
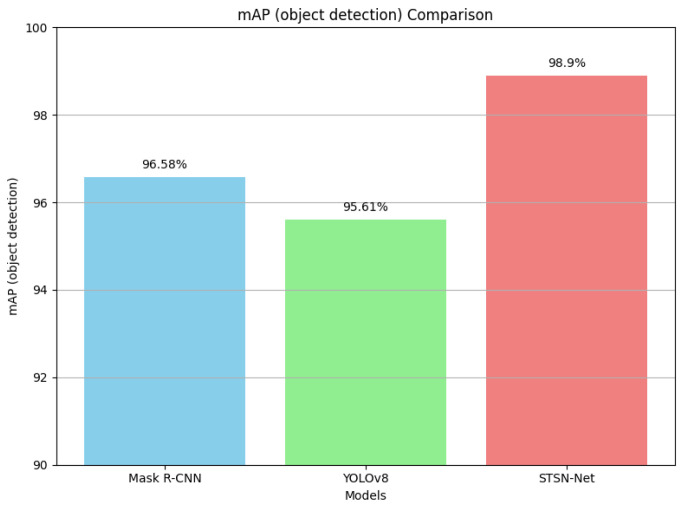
Comparison of mAP (object detection) results.

The mAP (object detection) results are illustrated in Figure 7 for clear representation.

**Figure 8:**
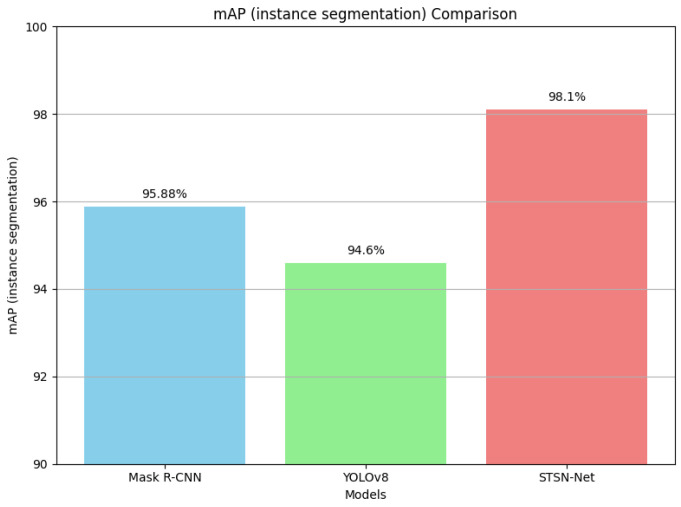
Comparison of mAP (instance segmentation) results.

The mAP (instance segmentation) results are illustrated in Figure 8 for clear representation.

**Figure 9:**
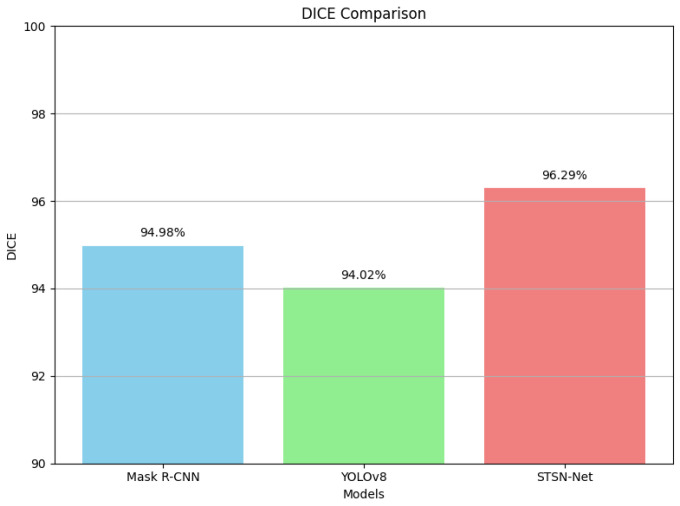
Comparison of DICE score.

The DICE score results are illustrated in Figure 9 for clear representation.

The inference times in milliseconds (ms) are depicted in Table 5.

## 4. Discussion

This study demonstrated the use of an STSN-Net framework comprising a modified DSN and an RSSN to obtain accurate segmentation and numbering of teeth in a crowded environment. The performances of the deformable convolution filter and dilated convolution filter in the DSN were analyzed. These filters ensured that different perspectives could be handled and that the receptive field was sufficiently large for a better representation of the panoramic X-ray images. The RSSN aided in determining the region with the highest probability of occurrence of the target while simultaneously reducing the rate of false positives. The STSN-Net architecture improved the performance of the detection task using information provided by the RSSN with an element-wise sum operation. Block I was a shared convolution block that extracted primary features, such as edges, lines, and corners. The extracted shared features were characterized as shallow features, which were generic in different networks and universally applicable for downstream tasks. These extracted features served as the building blocks for more complex patterns in the subsequent DSN and RSSN, which enhanced the feature reusability and reduced the model size. The inclusion of Block I in the network architecture facilitated initial data processing and ensured that downstream blocks received preprocessed, feature-rich inputs. This strategy not only streamlined the overall computational process but also enhanced the ability of the network to generalize across different tasks by providing a versatile feature set. Block II was the modified DSN that employed the deformable convolution filter and dilated convolution filter to handle various perspectives of the target and provided a large receptive field to understand the contextual information around the target. Block III was the RSSN that simultaneously processed the shared feature maps. Multiscale features were extracted and fused in a specific manner. A 3 × 3 convolution filter was used to extract the features while maintaining a high resolution. Features of different scales were fused using max-pooling, and up-convolution was performed depending on the source and target resolution. A skip connection was employed to make full use of the features at each resolution while reducing the number of network parameters. The RSSN provided useful information on the spatial location of the target and not only optimized the quality of the proposed regions obtained from the DSN but also minimized the background noise. Overall, the STSN-Net framework provided excellent performance for multiple tasks, and the F1 score of the detection task was 98.49% (best F1 score), the mAP (object detection) score was 98.90%, the mAP (instance segmentation) score was 98.10%, and the DICE score was 96.29%, which outperformed the two state-of-the-art models (Mask RCNN with Swin Transformer backbone network and the YOLOV8 for instance segmentation).

In addition to the aforementioned features of the STSN-Net framework, it is crucial to address the challenge of class imbalance in dental imaging, notably, the less frequent occurrence of certain tooth types. Class imbalance is a significant concern in medical imaging, as it can skew the performance of the model, leading to suboptimal detection and segmentation of less-represented classes. The proposed STSN-Net framework tackled this challenge in multiple ways. First, the modified DSN, with its deformable and dilated convolution filters, was adept at handling varying perspectives and tooth morphologies. This flexibility ensured that even less common tooth types were accurately captured and represented, thus mitigating the impact of class imbalance on the performance of the model.

Furthermore, the ability of RSSN to focus on regions with the highest probability of target occurrence played a pivotal role in addressing class imbalance. By prioritizing the regions likely to contain the target, the RSSN effectively enhanced the detection of underrepresented tooth types. This selective focus, combined with the reduction of false positives, ensured a balanced treatment of all tooth classes in the segmentation and numbering process.

Additionally, the multiscale feature extraction and fusion in the RSSN contributed to this balance. By extracting and combining features at different scales, the network ensured high-resolution representations of all tooth types regardless of their frequency in the dataset. The use of skip connections further guaranteed that features from each resolution were fully utilized, thereby augmenting the sensitivity of the network to less frequent tooth types.

Furthermore, most existing studies in dental arch classification rely on bounding box annotations without segmenting teeth based on their morphology. Additionally, samples with significant dental crowding are often excluded, which leads to reduced practicality in clinical application. In contrast, our study utilized a dataset comprising panoramic radiographs across various age groups. This dataset encompassed common pathological imaging features encountered in clinical practice, such as deciduous teeth, residual crowns, roots, restorations, root canal treatments, periapical lesions, impacted teeth, implants, brackets, and titanium plates. Because of the inclusion of these features, our model adeptly handled various complex clinical scenarios, thus enhancing its effectiveness in segmentation and tooth position recognition.

The application of artificial intelligence in automatic segmentation and identification of teeth in panoramic radiographs has considerably boosted diagnostic efficiency across various dental specialties. Rapid tooth position identification has assisted clinicians in diagnosing the number and location of missing teeth, thus preventing diagnostic errors, aiding in medical decision-making, and facilitating the documentation of case histories. In large-scale epidemiological surveys, automated tooth position recognition has permitted the analysis of extensive panoramic radiograph data, providing valuable insights into the oral health status of specific populations at a reduced cost.

Moreover, rapid automatic tooth segmentation serves as the foundation for various AI-assisted diagnostic models. By accurately identifying the tooth morphology, the crown–root ratio can be calculated, the completeness of tooth crowns can be ascertained, the type of impaction in wisdom teeth can be determined, and alveolar bone height can be assessed. In instances of radiographic overlap between teeth, segmentation aids in distinguishing pathological issues or restorations specific to each tooth. Moreover, segmenting the alveolar ridge line enables the quick assessment of alveolar bone height and, combined with tooth segmentation results, facilitates the diagnosis of periodontal conditions.

## 5. Conclusions

This study has described a unique STSN-Net architecture for the accurate segmentation and numbering of teeth with a best F1 score of 98.49%, an mAP score of 98.10%, and a DICE score of 96.29%, which is well suited for use in oral clinical practice and has the potential to assist doctors. This method can help doctors obtain an objective and accurate segmentation and numbering of the teeth and standardize the oral clinical process while simultaneously reducing their workload. The proposed STSN-Net framework for automatic segmentation and tooth numbering in panoramic radiographs marks a significant advancement in augmenting diagnostic and therapeutic efficiency across various dental specialties. Furthermore, in the context of large-scale epidemiological studies, the proposed STSN-Net offers a promising avenue to analyze extensive datasets of panoramic radiographs. Such automation aids in acquiring comprehensive oral health data for specific populations at a reduced cost. In the future, the performance of this method should be evaluated in more clinical practices to further validate the application potential of the proposed framework.

## Figures and Tables

**Table 1:** Details of the dataset split.

Category	Train	Validation	Test	Total
Permanent Dentition	1115	228	228	1571
Primary Dentition	422	86	87	595
Total	1500	300	316	2116

**Table 2:** Distribution of the dataset.

Subcategory	Train	Validation	Test	Total
Missing Teeth	427	87	88	602
Dental Crowding	837	171	172	1180
Impacted Teeth	528	108	108	744
Teeth with Fillings	384	78	79	541
Teeth with Restorations	328	67	67	462

**Table 3:** *F*1 score obtained by the baseline, Mask R-CNN (using Swin Transformer as backbone network), YOLOv8 and the proposed network (contributions of different steps of the proposed network were also demonstrated).

Base Size (1333 × 800)	F1 (1.0×)	F1 (1.2×)	F1 (1.4×)	F1 (1.6×)
Baseline	97.36%	97.53%	97.64%	97.88%
+ Deformable convolution	98.09%	98.14%	98.19%	98.23%
+ Dilated convolution	98.08%	98.13%	98.14%	98.16%
+ Region segmentation	98.11%	98.12%	98.15%	98.22%
Mask R-CNN	97.49%	97.62%	97.82%	97.89%
YOLOv8	96.49%	96.78%	96.89%	97.02%
The proposed network	98.30%	98.32%	98.38%	98.49%

**Table 4:** mAP and DICE scores obtained by the Mask R-CNN (using Swin Transformer as backbone network), YOLOv8, and the proposed network.

Metrics	Mask R-CNN	YOLOv8	STSN-Net
mAP (object detection)	96.58%	95.61%	98.90%
mAP (instance segmentation)	95.88%	94.60%	98.10%
DICE	94.98%	94.02%	96.29%

**Table 5:** Inference times of the baseline and different steps of the proposed method (the numbers represent the average inference time per image).

Base Size (1333 × 800)	F1 (1.0×)	F1 (1.2×)	F1 (1.4×)	F1 (1.6×)
Baseline	85	89	94	100
+ Deformable convolution	100	110	115	120
+ Dilated convolution	95	98	100	110
+ Region segmentation	90	95	99	104
The proposed network	110	115	118	121

## Data Availability

The data presented in this study are available on request from the corresponding author.
